# A Lignin-Based Zwitterionic Surfactant Facilitates Heavy Oil Viscosity Reduction via Interfacial Modification and Molecular Aggregation Disruption in High-Salinity Reservoirs

**DOI:** 10.3390/molecules30112419

**Published:** 2025-05-31

**Authors:** Qiutao Wu, Tao Liu, Xinru Xu, Jingyi Yang

**Affiliations:** 1 International Joint Research Center of Green Energy Chemical Engineering, East China University of Science and Technology, Meilong Road 130, Shanghai 200237, China; w117333094@163.com (Q.W.); xrxu86@ecust.edu.cn (X.X.); 2 Shanghai Key Laboratory of Multiphase Materials Chemical Engineering, School of Chemical Engineering, East China University of Science and Technology, Shanghai 200237, China; liutao@ecust.edu.cn

**Keywords:** lignin-based surfactants, heavy oil emulsification, high-salinity reservoirs, molecular dynamics simulation, green surfactant

## Abstract

The development of eco-friendly surfactants is pivotal for enhanced oil recovery (EOR). In this study, a novel lignin-derived zwitterionic surfactant (DMS) was synthesized through a two-step chemical process involving esterification and free radical polymerization, utilizing renewable alkali lignin, maleic anhydride, dimethylamino propyl methacrylamide (DMAPMA), and sulfobetaine methacrylate (SBMA) as precursors. Comprehensive characterization via ^1^H NMR, FTIR, and XPS validated the successful integration of amphiphilic functionalities. Hydrophilic–lipophilic balance (HLB) analysis showed a strong tendency to form stable oil-in-water (O/W) emulsions. The experimental results showed a remarkable 91.6% viscosity reduction in Xinjiang heavy crude oil emulsions at an optimum dosage of 1000 mg/L. Notably, DMS retained an 84.8% viscosity reduction efficiency under hypersaline conditions (total dissolved solids, TDS = 200,460 mg/L), demonstrating exceptional salt tolerance. Mechanistic insights derived from zeta potential measurements and molecular dynamics simulations revealed dual functionalities: interfacial modification by DMS-induced O/W phase inversion and electrostatic repulsion (zeta potential: −30.89 mV) stabilized the emulsion while disrupting π–π interactions between asphaltenes and resins, thereby mitigating macromolecular aggregation in the oil phase. As a green, bio-based viscosity suppressor, DMS exhibits significant potential for heavy oil recovery in high-salinity reservoirs, addressing the persistent challenge of salinity-induced inefficacy in conventional chemical solutions and offering a sustainable pathway for enhanced oil recovery.

## 1. Introduction

Heavy oil resources are abundant worldwide, accounting for approximately 70% of total crude oil reserves, and are considered a vital alternative to conventional oil sources [1,2]. However, heavy oil poses inherent challenges such as high viscosity and poor flowability, which hinder its extraction and transportation [3]. Industrial practice relies primarily on thermal recovery methods (e.g., steam-assisted gravity drainage (SAGD) and in situ combustion (ISC)) [4,5]. In addition to thermal methods, several non-thermal techniques have been proposed to reduce heavy oil viscosity. These include dilution with light hydrocarbons (e.g., naphtha or condensates) [6,7], heavy oil catalysis [8,9], and the application of various chemical agents such as surfactants, dispersant viscosity reducer [10,11], or nanoparticles [12,13]. Among these, emulsification-based chemical flooding has gained considerable attention in recent years. This method involves the formation of oil-in-water emulsions using surfactants to reduce viscosity and improve flowability, offering a promising alternative to thermal recovery—particularly for conventional heavy oils with viscosities ranging from 50 to 5000 mPa·s. Compared to thermal methods, emulsification can enhance oil recovery while significantly lowering energy input and carbon emissions, as it avoids the need for thermal stimulation. The emulsification–viscosity reduction in heavy oil has been extensively studied, with a variety of surfactants being evaluated for their effectiveness. Table 1 presents part of the results obtained in this study.

Industrial practices have identified certain disadvantages associated with anionic, cationic, and non-ionic surfactants. Specifically, anionic surfactants (e.g., sulfonates and sulfates) tend to adsorb strongly in acidic environments and are incompatible with divalent cations [22,23]. Cationic surfactants, such as cetyltrimethylammonium bromide, are costly and exhibit high adsorption on negatively charged sandstone surfaces, which limits their use in conjunction with anionic additives [24,25]. Nonionic surfactants (e.g., alkyl polyglucosides) suffer from poor solubility at low temperatures and limited thermal stability [26]. In contrast, zwitterionic surfactants—such as sulfobetaines—have attracted increasing interest in enhanced oil recovery (EOR) applications due to their outstanding salt and temperature tolerance, synergistic performance, and favorable solubility profiles [27]. Their physicochemical properties are highly sensitive to molecular structure, prompting ongoing efforts to optimize both hydrophobic tail groups and hydrophilic head moieties [28,29]. Surfactants containing aromatic rings have shown enhanced surface activity. For instance, Gao et al. synthesized alkyl and phenyl-containing betaines, finding that phenyl substitution halved the critical micelle concentration (CMC) and reduced interfacial tension by two orders of magnitude compared to alkyl analogs [30]. Similarly, Zuo et al. developed aromatic carboxybetaine and sulfobetaine surfactants with improved salt tolerance, capable of reducing the interfacial tension between Xinjiang crude oil and water to the 10^−3^ mN/m range under alkali-free conditions [31]. However, these hydrophobic groups are typically petroleum-derived, raising concerns about sustainability and production cost. There is therefore an urgent demand for renewable alternatives.

Lignin, a highly promising bio-based material, is abundant, cost-effective, and rich in reactive functionalities—such as aryl, hydroxyl, carbonyl, and carboxyl groups—which enable diverse chemical modifications, including esterification, etherification, sulfonation, and reductive amination [32,33,34]. Currently, lignin has achieved certain results in fields such as energy production [35], dye dispersants [36], UV absorbers [37,38], phenolic resins [38], and petroleum [39]. In recent years, lignin-based surfactants have garnered growing attention from researchers, with preliminary progress achieved in molecular design and performance evaluation. Chen et al. have developed anionically modified nonionic lignin surfactants [40] and Gemini bis-sulfonate lignin derivatives [41] through alkaline lignin modification, which achieved ultralow oil–water interfacial tension levels. However, there has been limited research on lignin-derived zwitterionic surfactants, particularly those capable of emulsifying heavy oil under extreme conditions.

To address this gap, a novel sustainable lignin-based zwitterionic surfactant (DMS) was synthesized via esterification and free radical polymerization, employing alkali lignin, maleic anhydride, DMAPMA, and SBMA. The chemical structure of DMS was systematically characterized by ^1^H NMR, FTIR, and XPS. Its salt tolerance, surface activity, and HLB were comprehensively evaluated. In parallel, the emulsification and viscosity reduction performance of Xinjiang heavy crude oil was assessed under varying conditions of temperature, pH, salinity, and oil–water ratio. Mechanistic insights into the emulsification and viscosity reduction processes were elucidated through zeta potential analysis, droplet size distribution, rheological measurements, and molecular dynamics simulations. This work offers a sustainable and efficient strategy for the development of lignin-derived zwitterionic surfactants, providing an eco-friendly solution for enhanced heavy oil recovery in saline reservoirs.

## 2. Results and Discussion

### 2.1. DMS Surfactant Structural Characterization

The molecular structure of DMS was confirmed through a combination of elemental analysis (Table 2) and gel permeation chromatography (GPC, Table 3). Elemental analysis revealed a sulfur content of 3.10 wt%, exclusively attributable to SBMA grafting. The nitrogen content (9.17 wt%) was found to originate from both DMAPMA and SBMA components. The grafting degree of SBMA (n) was calculated as 3.52 based on sulfur content. Subsequent analysis of nitrogen content after deducting the contribution from SBMA yielded a DMAPMA grafting degree (m) of 10.16. The theoretical molecular weight increase, derived from grafting degrees and monomer molecular weights, was calculated to be 2714 g/mol. This value is in excellent agreement with the molecular weight increase of 2612 g/mol obtained from GPC experiments, showing only a minor 3.8% discrepancy (GPC chromatogram shown in Figure S1 of the Supplementary Materials).

**Figure 1:**
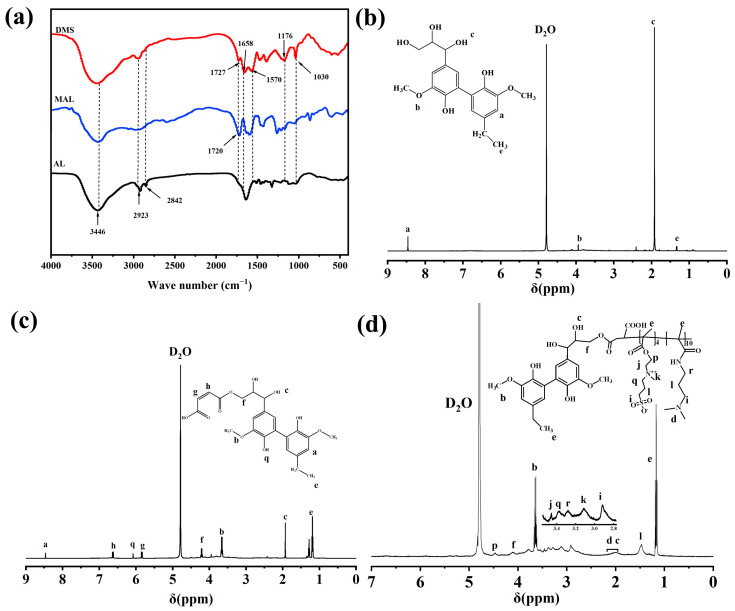
Characterization of products at different stages. (**a**) FT-IR spectra of AL, MAL, and DMS; (**b**) ^1^H NMR spectrum of AL; (**c**) ^1^H NMR spectrum of MAL; (**d**) ^1^H NMR spectrum of DMS.

The molecular structure of DMS was systematically validated through a combination of ^1^H NMR, FTIR, and XPS analyses. As demonstrated in Figure 1a, the FTIR spectrum of AL displays characteristic bands: a broad peak at 3446 cm^−1^ attributed to phenolic -OH stretching vibrations [42], and C-H stretching modes at 2923/2842 cm^−1^ corresponding to -CH_3_ and -CH_2_ groups. Following maleic anhydride modification, the MAL spectrum exhibits a prominent ester carbonyl (C=O) stretching vibration at 1720 cm^−1^, confirming successful esterification [43]. In the final DMS product, critical functional groups were identified: (i) amide functionalities: C=O stretching (1658 cm^−1^, amide I) [44] and coupled N-H bending/C-N stretching (1570 cm^−1^, amide II), confirming DMAPMA incorporation. S=O symmetric (1030 cm^−1^) and asymmetric (1176 cm^−1^) stretching vibrations verifying SBMA grafting [45]. This spectral evolution—from AL to MAL to DMS—provides definitive evidence of progressive structural modification.

^1^H NMR analysis was performed on AL, MAL, and DMS. As shown in Figure 1b, the spectrum of AL exhibits the following resonances: aromatic protons of benzene rings at δ 8.46 ppm (a), methoxy group protons (-OCH_3_) at δ 3.95 ppm (b), protons adjacent to aliphatic hydroxyls at δ 1.93 ppm (c), and aliphatic -CH_3_ protons on lignin side chains at δ 1.33 ppm (e). The MAL spectrum (Figure 1c) displays vinyl protons (-CH=CH-) from grafted maleic anhydride at δ 6.7 ppm (h) and δ 5.8 ppm (g), with methylene protons adjacent to vinyl groups appearing at δ 4.1 ppm (f) [43]. For DMS (Figure 1d), the characteristic peaks are assigned as follows: δ 4.48 ppm (p) corresponds to the -COOCH_2_- group, δ 3.12 ppm (k) is attributed to the -N^+^(CH_3_)_2_ group, and signals at δ 3.45 ppm (j) and δ 3.37 ppm (q) are assigned to -CH_2_- moieties adjacent to the quaternary ammonium group; all of these signals originate from SBMA [46]. Additional resonances at δ 2.1 ppm (d) and δ 3.2 ppm (r) indicate dimethylamino and amide-linked -CH_2_- units, respectively, both derived from DMAPMA [47]. The remaining signals include δ 1.52 ppm (l) (central -CH_2_- in grafted monomers) and δ 2.9 ppm (i) (-CH_2_- proximal to sulfonate and dimethylamino functionalities). Critically, the complete absence of vinyl proton signals confirms successful double bond consumption during the reaction.

**Figure 2:**
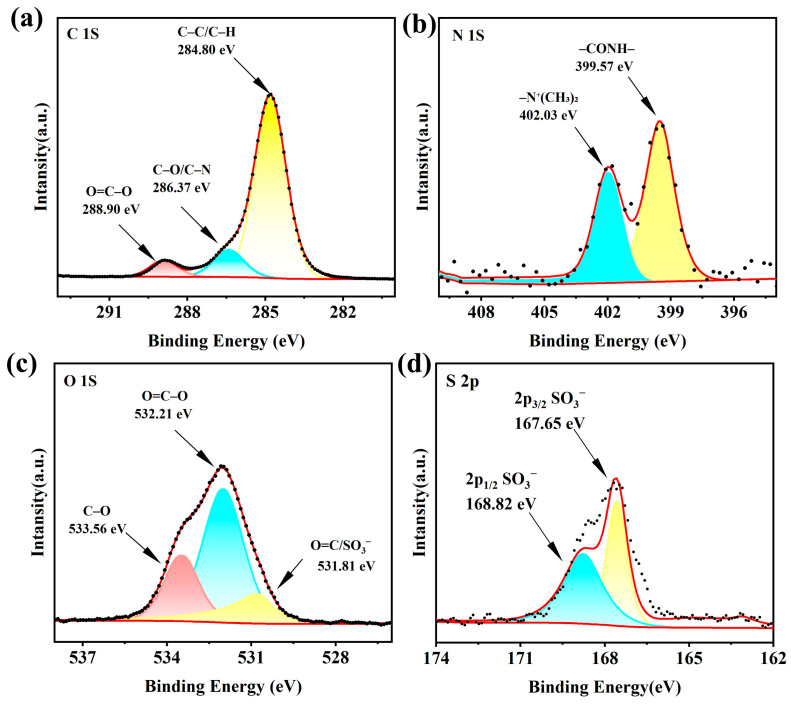
XPS characterization analysis of DMS surfactant. (**a**) C 1s spectrum; (**b**) N 1s spectrum; (**c**) O 1s spectrum; (**d**) S 2_p_ spectrum.

X-ray photoelectron spectroscopy was employed to further validate the molecular architecture of DMS. High-resolution spectroscopic deconvolution reveals critical bonding configurations (Full spectrum in Figure S2 of the Supplementary Materials). The C 1s spectrum (Figure 2a) is as follows: the spectrum contains three resolved peaks corresponding to aliphatic carbons (C–C/C–H, 284.8 eV), ether/amine-linked carbons (C–O/C–N, 286.37 eV), and ester/carboxylate carbons (O=C–O, 288.90 eV) [44]. In the N 1s spectrum (Figure 2b), two nitrogen environments are evident: amide nitrogen (–CONH– at 399.57 eV) from DMAPMA and quaternary ammonium nitrogen (–N^+^(CH_3_)_2_ at 402.03 eV) from SBMA [48]. The O 1s spectrum (Figure 2c) shows that the spectrum is composed of three distinct components at 531.81 eV (sulfonate/carbonyl oxygen, SO3-/C=O), 532.21 eV (ester oxygen, O=C-O), and 533.56 eV (ether oxygen, C-O) [49]. The S 2p spectrum (Figure 2d) displays a characteristic doublet with S 2p3/2 (167.65 eV) and S 2p1/2 (168.82 eV), showing a spin-orbit splitting of 1.17 eV and an area ratio of 2:1, consistent with sulfonate group (SO_3_^−^) electronic states. These spectral assignments confirm the integration of SBMA and DMAPMA moieties into the lignin-based surfactant structure.

**Figure 3:**
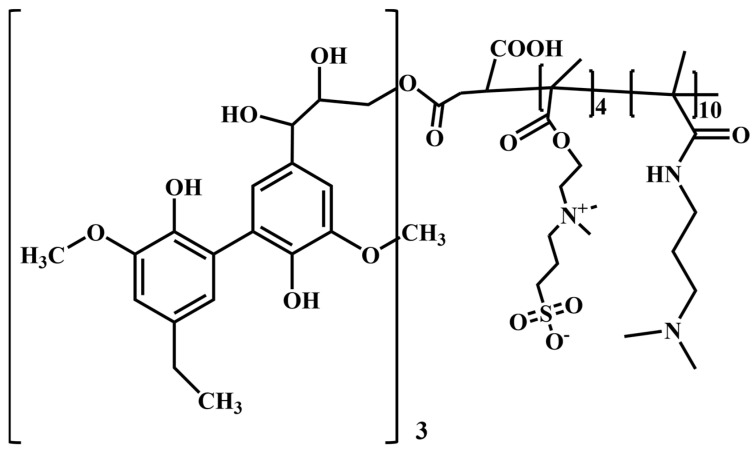
Structure of DMS surfactant.

In summary, a thorough analysis of the structure of the target product, DMS surfactant, was conducted. Elemental analysis and GPC calculations indicated approximately 4 SBMA units (n = 4) and 10 DMAPMA units (n = 10). The molecular structure of the DMS surfactant is presented in Figure 3. Due to the complex and broad molecular weight distribution of AL, minimum structural units were used to represent the AL molecules. Each minimum structural unit has a molecular weight of 354, and each lignin molecule contains approximately three such units.

### 2.2. DMS Surfactant Performance

#### 2.2.1. HLB Values of DMS Surfactants

**Figure 4:**
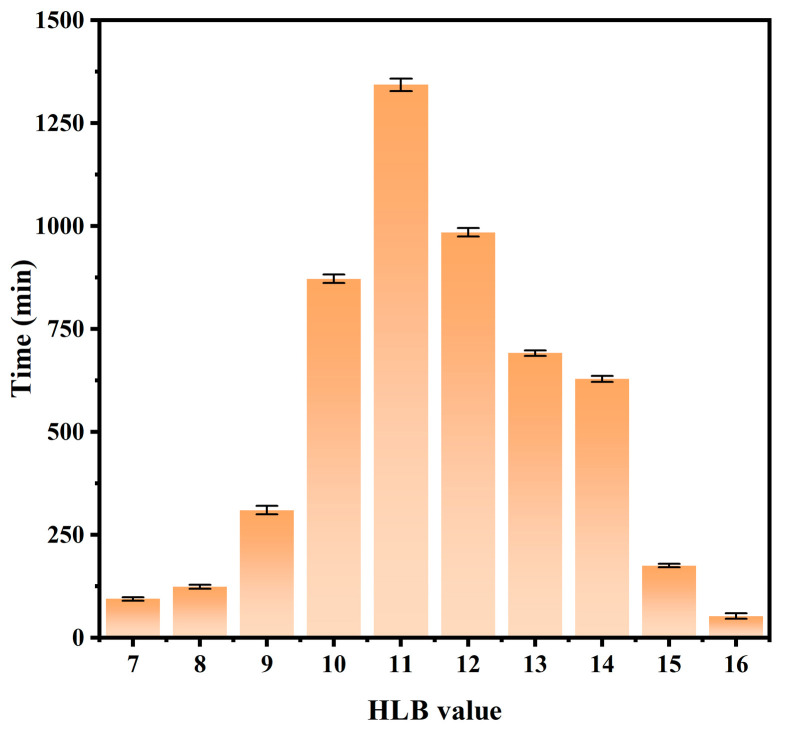
HLB value of the DMS surfactant.

Surfactants are amphiphilic molecules composed of hydrophilic and lipophilic groups, with their HLB value commonly used to quantify the relative proportion between these functionalities. A lower HLB value corresponds to stronger lipophilicity, while a higher value indicates greater hydrophilicity. As a semi-empirical parameter, the HLB value serves as an important guideline for surfactant selection in EOR applications [50]. Figure 4 illustrates the temporal progression of oil–water separation for DMS surfactant in emulsions exhibiting varying HLB values. The experimental measures determined the HLB value of DMS to be 11. Falling within the optimal range of 8–13, DMS effectively promotes the formation of stable O/W emulsions, consistent with the established relationship between surfactant physicochemical properties and HLB values [51].

#### 2.2.2. Surface Tension

**Figure 5:**
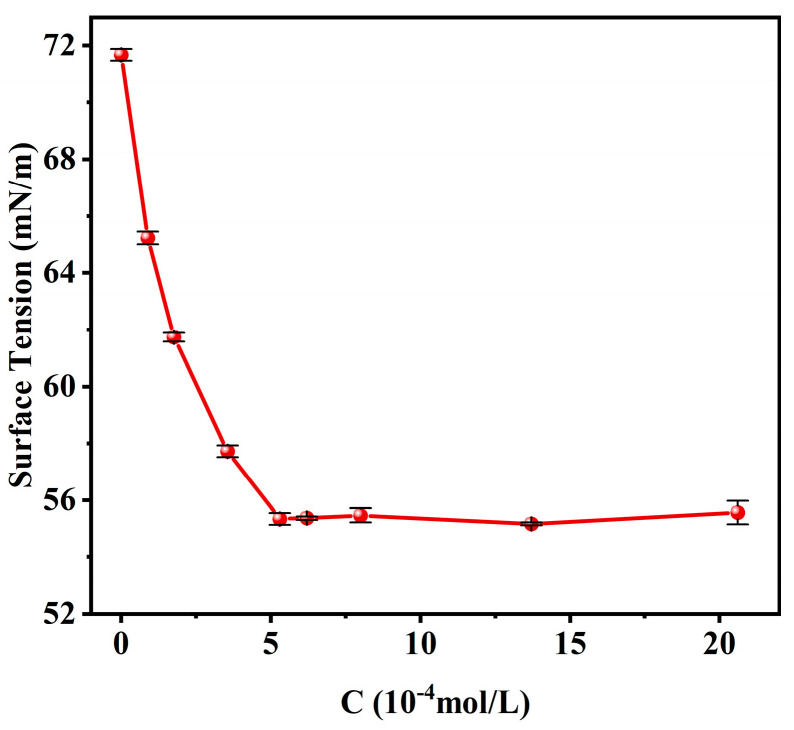
Surface tension versus surfactant concentration.

The CMC of a surfactant influences its emulsification efficiency; above the CMC, micelle formation enhances emulsification and contributes to viscosity reduction in heavy oil. The surface tension of DMS and Sodium dodecylbenzene sulfonate (SDBS) was measured at 25.0 °C using the Du Noüy ring method. As demonstrated in Figure 5, the surface tension–concentration curve(γ-c) curve exhibits two distinct regions: a linear decrease (c < CMC) attributable to monolayer adsorption at the air–water interface, and a plateau (c ≥ CMC) indicative of micellization. The CMC, determined from the inflection point, was found to be 5.3 × 10^−4^ mol/L, which is significantly lower than the CMC of SDBS (2.10 × 10^−3^ mol/L), as reported in [52]. This outcome demonstrates that DMS exhibits enhanced interfacial activity and micellization efficiency at low concentrations.

#### 2.2.3. Salt Tolerance

**Figure 6:**
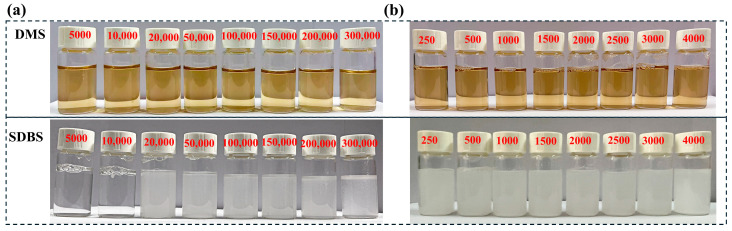
Precipitation conditions of DMS and SDBS under different salt concentrations. (**a**) NaCl concentration (mg/L); (**b**) CaCl_2_ concentration (mg/L).

Surfactant salt tolerance critically determines their viability in high-salinity reservoirs. Comparative stability tests under simulated salinity gradients (NaCl: 5000–300,000 mg/L; CaCl_2_: 250–4000 mg/L) revealed DMS’s superior performance. DMS maintained colloidal stability across all tested conditions, while SDBS precipitated at NaCl: ≥ 20,000 mg/L and CaCl_2_ ≥ 250 mg/L in Figure 6. This contrast stems from fundamental structural differences. The linear sulfonate groups in SDBS undergo ion pairing with NaCl/CaCl_2_, inducing precipitation. In contrast, the salt tolerance mechanism of DMS arises from two synergistic effects: (1) the three-dimensional lignin network structure provides steric stabilization against ionic perturbations [53]; and (2) the intrinsic charge-balanced configuration of the oligomeric surfactant enables electrostatic self-regulation, effectively preventing precipitation [54].

### 2.3. Evaluation of Emulsification and Viscosity Reduction Performance of DMS Surfactant

Unless otherwise specified, all subsequent evaluations were conducted under standardized conditions: a temperature of 50 °C, an oil–water ratio of 6:4, a surfactant concentration of 1000 mg/L, and a water salinity of 10,023 mg/L.

#### 2.3.1. Effects of Different Types of Surfactants on Viscosity of Heavy Oil Emulsification

**Figure 7:**
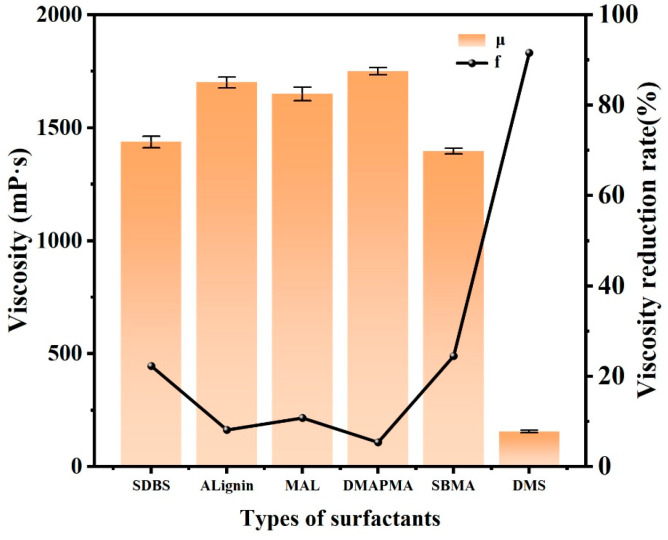
Effects of different surfactants on viscosity reduction rates of heavy oil emulsion.

The viscosity reduction performance of various surfactants—including SDBS, alkali lignin (AL), MAL, DMAPMA, SBMA, and the synthesized DMS—on Xinjiang heavy crude oil emulsions is shown in Figure 7. Among these, DMS exhibited the highest efficiency, achieving a viscosity reduction rate of 91.6%, which was markedly superior to that of SDBS (22.3%), AL (8.1%), MAL (10.8%), DMAPMA (5.4%), and SBMA (24.5%). The moderate surface activity of AL stems from its aromatic rings and polar groups, such as hydroxyl and carboxyl. However, its effectiveness in emulsifying and reducing the viscosity of heavy oil is limited due to its complex molecular structure. The large molecular weight, abundant aromatic rings, and unevenly distributed polar groups lead to poor hydrophilic–lipophilic balance and hinder its alignment at the oil–water interface. Moreover, AL tends to aggregate in neutral or weakly acidic conditions, resulting in poor dispersibility and an inability to form stable emulsions or colloidal systems [55,56]. To overcome these limitations, anionic and cationic monomers—SBMA and DMAPMA—were introduced into the AL framework. This structural modification significantly improved the solubility and interfacial activity of the resulting DMS molecule. The enhanced molecular configuration facilitated effective adsorption at the oil–water interface and promoted phase inversion from a water-in-oil (W/O) to an O/W emulsion system, thereby dramatically enhancing viscosity reduction performance.

#### 2.3.2. Effect of the Concentration of DMS Surfactant on the Emulsification and Viscosity Reduction Effect of Heavy Oil

**Figure 8:**
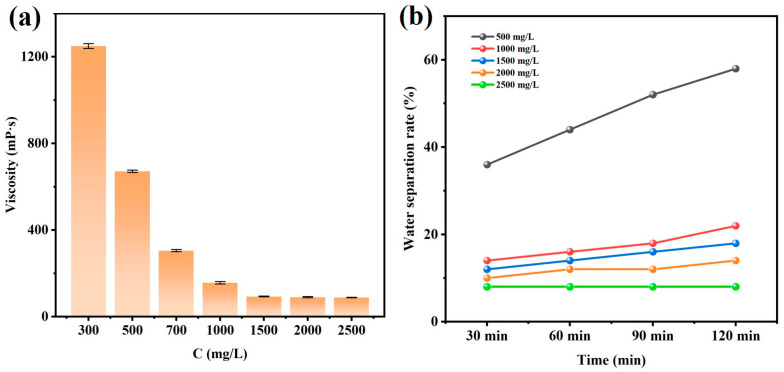
Effects of DMS surfactant content on viscosity reduction rate (**a**) and water separation rate (**b**) of heavy oil emulsions.

The effect of DMS surfactant concentration on the viscosity of heavy oil emulsions is evident in Figure 8a. It can be observed that as the concentration increases, the viscosity decreases. This phenomenon can be attributed to the surfactant’s adsorption behavior at the oil–water interface: at low concentrations, the interface adsorption is insufficient, and the emulsification effect is limited; as the concentration increases, the interface adsorption gradually becomes saturated, forming a dense interfacial film that significantly reduces the viscosity of the system [57,58]. Once a certain concentration is reached, the adsorption of the interfacial film gradually reaches equilibrium, and the viscosity reduction rate stabilizes. When the DMS concentration is 1000 mg/L, the viscosity reduction rate reaches 91.6%, demonstrating the effectiveness of DMS surfactants in reducing heavy oil viscosity.

As shown in Figure 8b, when the DMS surfactant concentration is below 500 mg/L, the water separation rate after 30 min reaches 36%, indicating that the heavy oil cannot be effectively emulsified at such low concentrations. In contrast, at a DMS concentration of 1000 mg/L, the 30 min water separation rate drops to 14%, and after standing for 120 min, it only increases slightly to 22%, suggesting a more stable emulsion. When the concentration increases further to 2500 mg/L, the 30 min water separation rate of the DMS emulsion decreases significantly to 8% and remains as low as 8% after 120 min of standing. These results indicate that in addition to reducing viscosity, increasing the DMS concentration also significantly enhances emulsion stability, effectively delaying phase separation and maintaining dispersion over time.

#### 2.3.3. Effect of pH on the Viscosity of Heavy Oil Emulsions

**Figure 9:**
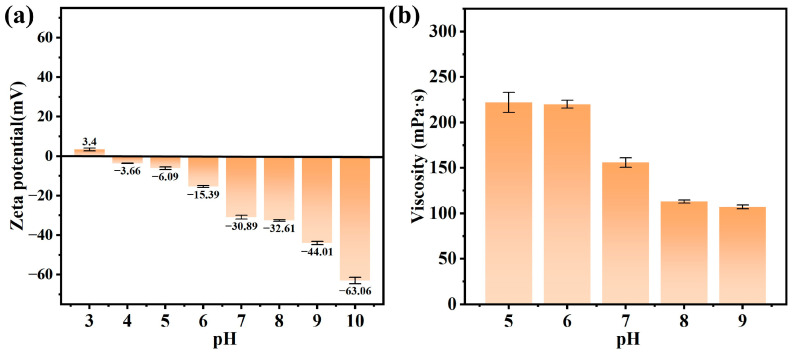
(**a**) Effect of pH on zeta potential of heavy oil; (**b**) effect of pH on the viscosity of heavy oil emulsions.

The pH-dependent variation in the zeta potential of heavy oil emulsions stabilized by the DMS surfactant is illustrated in Figure 9a. As the pH increases from 3 to 10, the zeta potential shifts from +3.4 mV to −63.09 mV. This behavior can be explained by examining both acidic and alkaline regimes. In the acidic range (pH < 7), the tertiary amine groups (-N(CH_3_)_2_) in the DMS surfactant are protonated, resulting in a positively charged surfactant. The cationic DMS molecules interact with negatively charged oil droplets, effectively neutralizing the surface charge and decreasing the absolute value of the zeta potential [53]. In contrast, in the neutral-to-alkaline range (pH ≥ 7), the deprotonation of the amine groups occurs, and ionization of functional groups such as phenolic hydroxyl and carboxyl groups leads to a net negative charge on the DMS molecules [59]. This results in a marked increase in the negative zeta potential, from −30.89 mV at pH 7 to −63.09 mV at pH 10, indicating enhanced electrostatic repulsion between droplets.

Figure 9b illustrates the influence of pH on the viscosity of DMS-stabilized heavy oil emulsions. The emulsion viscosity exhibited a progressive decrease from 222 mPa·s (pH 5) to 107 mPa·s (pH 9). A cross comparison with Figure 9a shows the mechanistic correlation: Under alkaline conditions (pH 9, |ζ| = 44.01 mV), the stabilization of the O/W emulsion occurs through enhanced electrostatic repulsion forces. This mechanism effectively suppresses droplet coalescence while maintaining a low viscosity continuous phase for optimum performance. Conversely, at a pH of 5, where |ζ| = 6.09 mV, the emulsion exhibited diminished electrostatic stabilization, leading to enhanced droplet coalescence and elevated viscosity.

#### 2.3.4. Effect of Salinity on the Viscosity of Heavy Oil Emulsions

**Figure 10:**
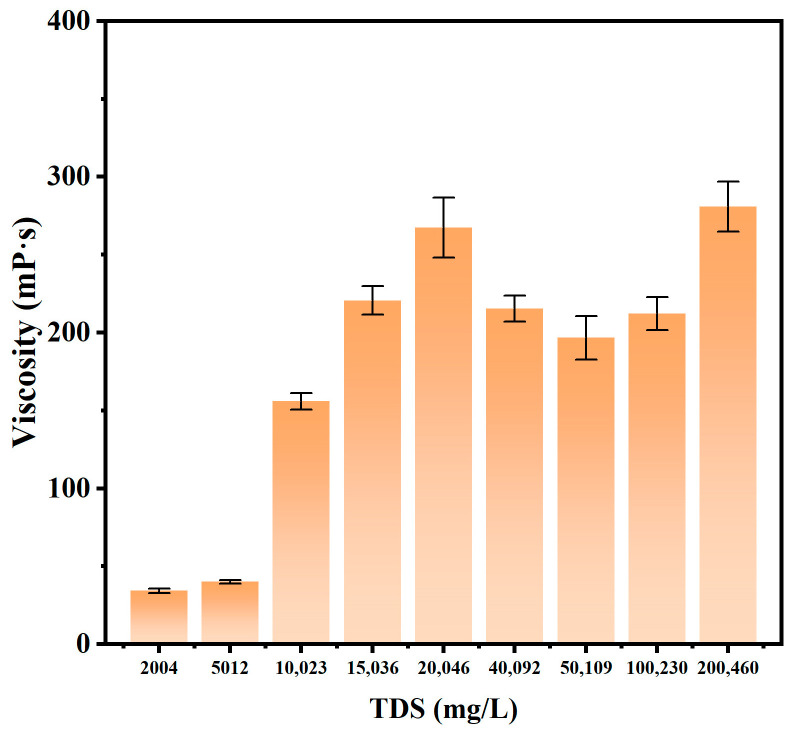
Effect of salinity on the emulsification and viscosity reduction in heavy oil.

The effect of salinity on the viscosity of heavy oil emulsions stabilized by the DMS surfactant is presented in Figure 10. The salinity in the experimental conditions ranged from 0.2 to 20 times the original formation water concentration. The emulsion viscosity increased from 34.3 mPa·s (0.2× salinity) to 281 mPa·s (20× salinity) with increasing salinity. Notably, the zwitterionic surfactant DMS maintained a >84.8% viscosity reduction efficiency over this salinity range. These results highlight the potential of DMS as a promising candidate for high-salinity reservoir conditions.

#### 2.3.5. Effect of Water Content on the Viscosity of Heavy Oil Emulsions

**Figure 11:**
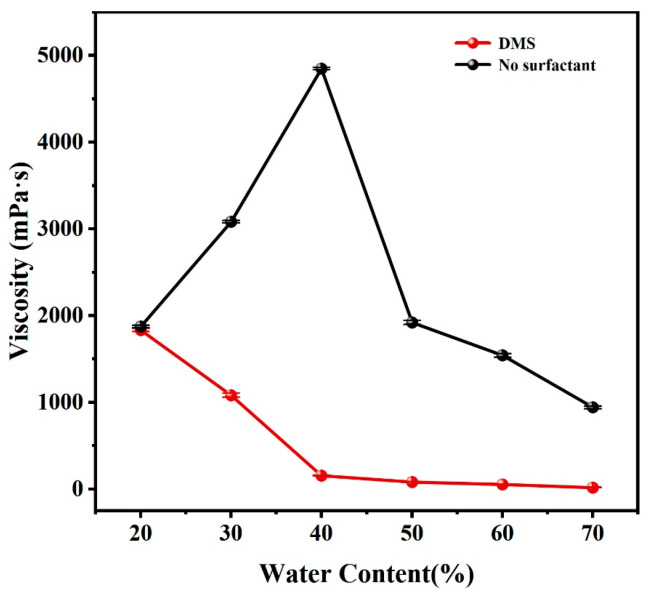
Effect of water content on heavy oil emulsion.

Figure 11 compares the viscosity reduction performance of heavy oil emulsions with and without the DMS surfactant across a range of water contents. In the absence of the surfactant, the emulsion viscosity reaches a maximum at 40% water content. At this point, due to the intensified interactions between water droplets, the fluid resistance also increases accordingly. Beyond this critical threshold, increased droplet collision frequency leads to coalescence, causing a sharp decline in viscosity. In contrast, the addition of the DMS surfactant significantly alters the rheological behavior of the heavy oil emulsion. At a water content of 20%, the viscosity remains comparable to that of pure heavy oil. This observation is consistent with the principles of close-packing theory [60], which suggests that when the volume fraction of the dispersed phase (i.e., water) is below 25.98%, phase inversion cannot occur, and the resulting emulsion remains in a W/O state. However, when the water content exceeds 30%, the presence of the DMS surfactant induces phase inversion, forming an O/W emulsion structure. These experimental findings demonstrate that DMS is effective in promoting emulsification of heavy oil even at relatively low water contents.

### 2.4. Viscosity Reduction Mechanism of Heavy Oil Emulsification

#### 2.4.1. Particle Size Distribution of Emulsions

**Figure 12:**
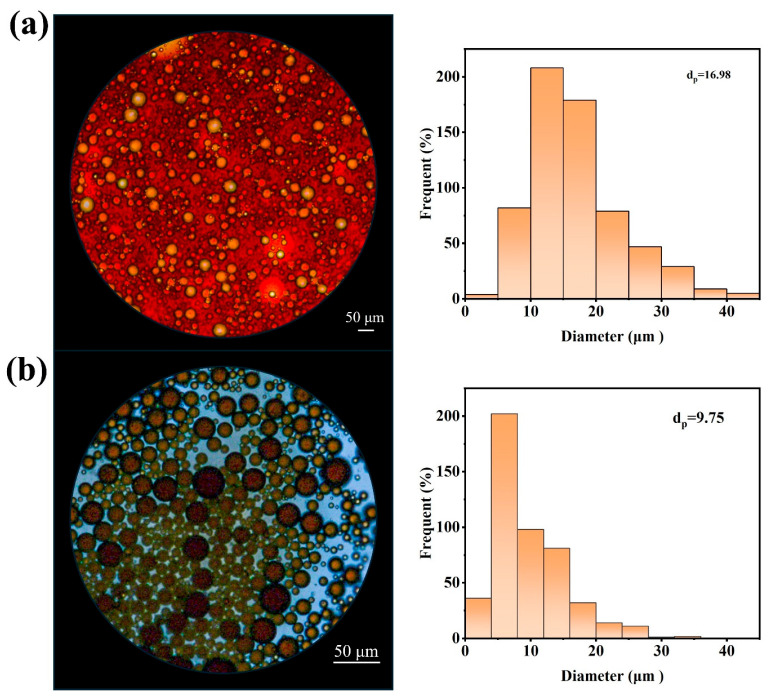
Micrographs and particle size distributions of heavy oil emulsions. (**a**) Without additives; magnification 10 times the objective. (**b**) With 1000 mg/L DMS; magnification 20 times the objective.

Figure 12 presents a comparative optical microscopy analysis of emulsions prepared under identical agitation conditions: (a) surfactant-free system (10× magnification) and (b) DMS-containing system (1000 mg/L, 20× magnification). In the absence of surfactant, a W/O emulsion formed, characterized by polydisperse water droplets (mean diameter: 16.98 μm) dispersed within the continuous oil phase. Remarkably, the introduction of DMS induced a complete phase inversion to an O/W emulsion, with monodisperse oil droplets (mean diameter: 9.75 μm) uniformly stabilized in the aqueous continuous phase. This transition is attributed to the interfacial activity of DMS, which reduced the oil–water interfacial tension and altered the HLB of the system. The significant reduction in droplet size and improved dispersion uniformity highlight DMS’s efficacy in enhancing emulsion stability through electrostatic repulsion mechanisms [17], as corroborated by zeta potential and rheological analyses.

#### 2.4.2. Oil–Water Interfacial Tension

**Figure 13:**
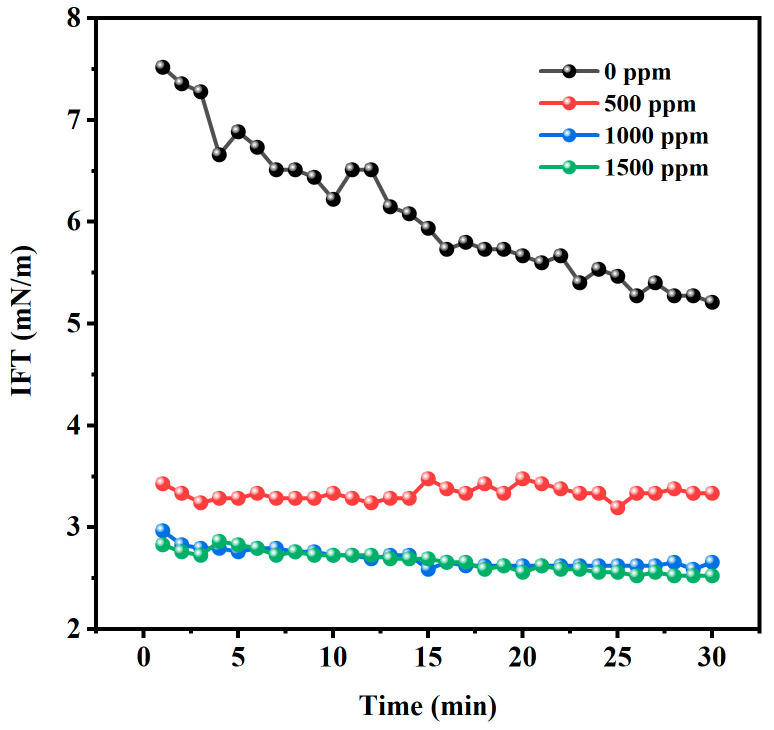
Effect of DMS surfactant concentration on oil–water interfacial tension.

The emulsification performance of a surfactant is intrinsically linked to its ability to reduce oil–water interfacial tension by modifying interfacial characteristics [61]. As illustrated in Figure 13, in the absence of an added surfactant, the oil–water interface is initially stabilized by indigenous surface-active components in heavy oil, predominantly asphaltenes. These high-molecular-weight amphiphilic molecules gradually adsorb at the interface, resulting in a time-dependent decrease in interfacial tension from an initial value of 7.52 mN/m to an equilibrium value of 5.20 mN/m. With the introduction of the DMS surfactant, the interfacial tension drops significantly due to the preferential and more efficient adsorption of DMS molecules at the oil–water interface. Increasing the DMS concentration further enhances this effect, with the interfacial tension reaching a minimum of 2.52 mN/m at 1000 mg/L. This reduction is attributed to the formation of a compact and resilient interfacial film, which replaces the loosely adsorbed asphaltenes and improves interfacial stability. However, as the concentration continues to increase beyond this point, the rate of interfacial tension reduction diminishes, indicating a saturation adsorption behavior. Once the interface becomes fully occupied by surfactant molecules, additional surfactant in the bulk phase exerts limited influence on interfacial properties. In conclusion, the DMS surfactant effectively reduces the oil–water interfacial tension, enhances interfacial film strength, and improves the compatibility between oil and water phases, thereby promoting the formation and stabilization of heavy oil emulsions.

#### 2.4.3. Zeta Potential at the Oil–Water Interface

**Figure 14:**
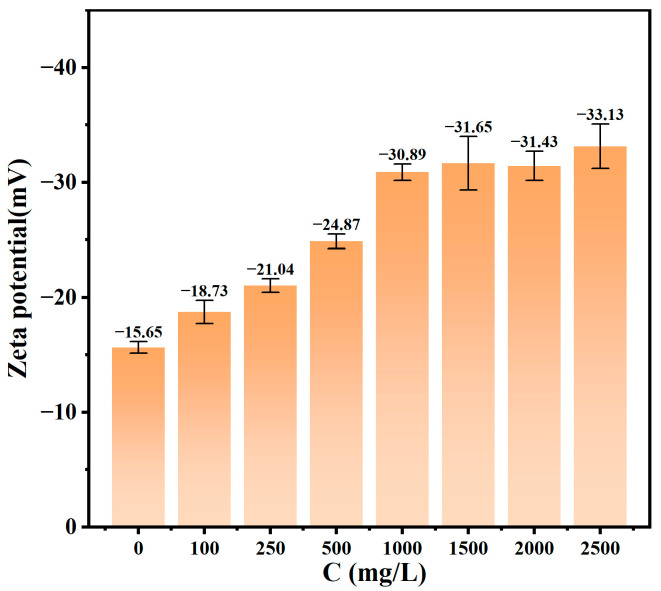
Effect of DMS surfactant on oil–water zeta potential.

As shown in Figure 14, the zeta potential of oil droplets dispersed in oil–water mixtures with DMS surfactant concentration at pH 7 exhibited an upward trend with increasing surfactant concentration. In the absence of a surfactant, the zeta potential registered −15.65 mV, an observation attributed to the presence of negatively charged acidic components (carboxylic acid and phenolic hydroxyl groups) that are intrinsic to crude oil particles. However, upon increasing the surfactant concentration to 1000 mg/L, a substantial rise in absolute zeta potential magnitude was observed, reaching 30.89 mV. It is noteworthy that at concentrations exceeding 1000 mg/L, the system exhibited |ζ| > 30 mV, indicative of enhanced electrostatic repulsion between oil droplets in O/W emulsions. This strengthening of interfacial charge has been demonstrated to be an effective means of suppressing droplet coalescence, thereby stabilizing the dispersed system [62].

#### 2.4.4. Rheological Properties

**Figure 15:**
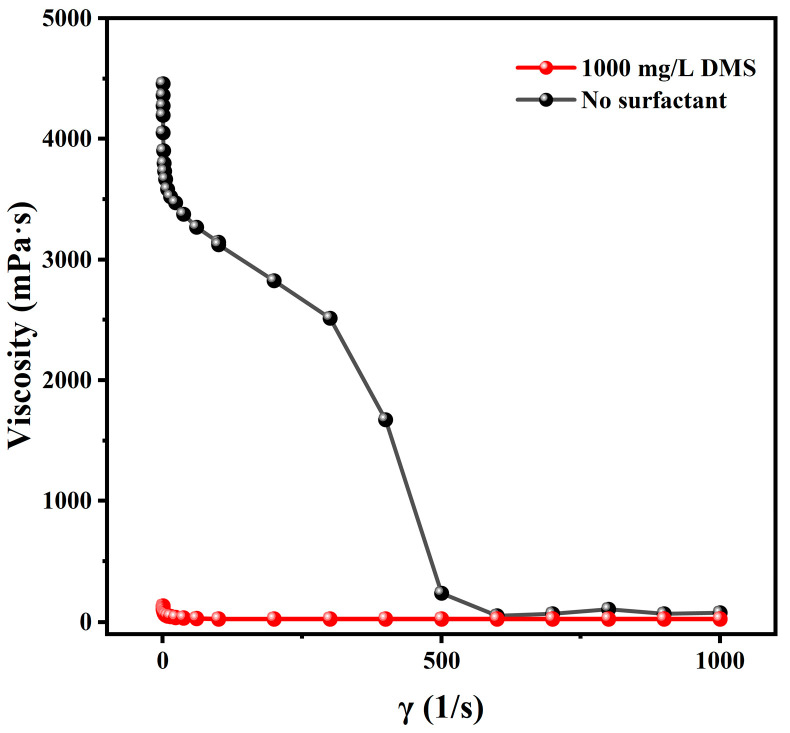
Effect of shear rate on rheological properties of oil–water emulsions before and after DMS addition.

As shown in Figure 15, the apparent viscosity of heavy oil emulsions evolves systematically under controlled experimental conditions. The surfactant-free W/O emulsion exhibited pronounced shear-thinning behavior, typical of non-Newtonian fluids, with viscosity decreasing by approximately 98.2% across the shear rate range. In contrast, the addition of DMS induced a phase inversion to an O/W emulsion, which displayed near-Newtonian flow characteristics. This transformation is primarily attributed to the increase in the zeta potential of dispersed oil droplets induced by DMS, which effectively suppresses droplet deformation and coalescence, thereby inhibiting the formation of interconnected network structures and promoting a more stable, flowable emulsion.

**Figure 16:**
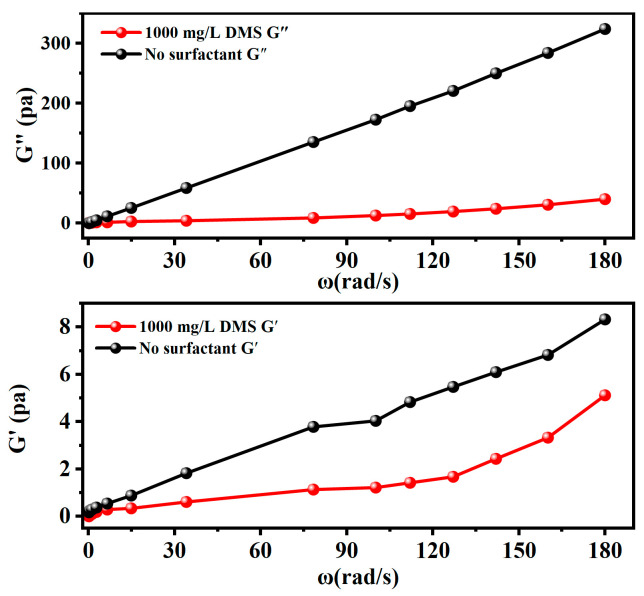
Effect of frequency on rheological properties of oil–water emulsions before and after DMS addition.

As shown in Figure 16, the frequency-dependent viscoelastic moduli—namely, the viscous modulus (G″) and elastic modulus (G′)—of heavy oil emulsions with and without DMS were compared under controlled conditions. Both systems exhibited viscosity-dominated behavior, as indicated by G″ consistently exceeding G′ across the entire frequency range. In the absence of DMS, a W/O emulsion was formed, with the continuous oil phase governing the viscoelastic response. Upon the addition of DMS, a phase inversion occurred, resulting in the formation of O/W emulsions. This inversion led to a decrease in the absolute values of both G″ and G′, attributable to the reduced viscosity of the aqueous continuous phase. These findings demonstrate that DMS enhances the flow properties of heavy oil emulsions by modifying interfacial structures and promoting the formation of more fluid, less elastic systems.

### 2.5. Molecular Dynamics Simulations

**Figure 17:**
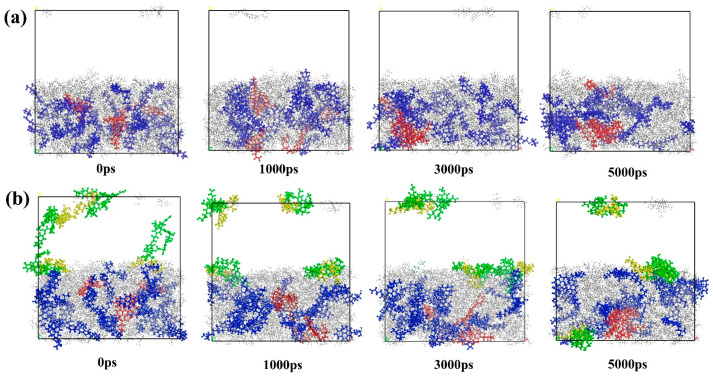
Snapshots of (**a**) surfactant-free systems and (**b**) systems with surfactant at 0 ps, 1000 ps, 3000 ps, and 5000 ps in the production phase simulation (water molecules omitted, red: asphaltenes, blue: resins, green: lipophilic groups in DMS, yellow: grafted monomer groups in DMS).

The molecular snapshots’ evolution at oil–water interfaces during 0–5000 ps is depicted for surfactant-free systems (Figure 17a) and DMS-modified systems (Figure 17b). In the absence of surfactants (Figure 17a), asphaltenes and resins gradually formed aggregates within the oil phase as the simulation time increased. This aggregation behavior enhances flow resistance, directly contributing to the high viscosity of heavy crude oils. In contrast, the DMS-modified system (Figure 17b) exhibited distinct interfacial reorganization. This was characterized by the spontaneous adsorption of DMS surfactant molecules at the oil–water interface as the simulation progressed. Concurrently, asphaltenes and resins in the oil phase accumulated in a looser structure.

**Figure 18:**
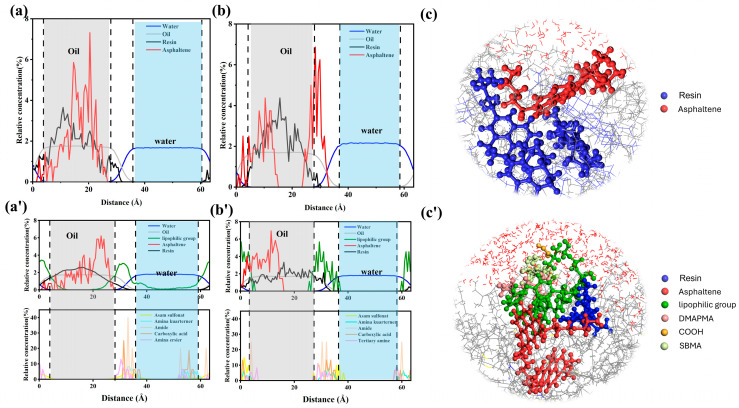
Relative concentration distribution of components along the z-axis within the system (the area inside the dotted line represents the oil–water interfacial film. transparent gray: oil phase, transparent blue: water phase). Surfactant-free systems: (**a**) 0 ps, (**b**) 5000 ps, (**c**) snapshots of oil–water interface at 5000 ps. Surfactant-containing systems: (**a′**) 0 ps, (**b′**) 5000 ps, (**c′**) snapshots of oil–water interface at 5000 ps.

The relative concentration distributions of components in the surfactant-free system are depicted at 0 ps and 5000 ps, respectively (Figure 18a,b). The oil–water interface was identified through concentration inflection points between the oil phase and the aqueous phase. The data were then subjected to normalization. With the prolongation of the simulation time, asphaltenes progressively adsorb at the oil–water interface while resin concentration increases within the oil phase. This divergence can be attributed to the higher polarity and interfacial activity of asphaltenes in comparison to resins, which leads to their preferential adsorption. Competitive adsorption displaces resins into the oil phase, elevating their relative concentration. Asphaltenes form aggregates with resins in the oil phase through π–π interactions and van der Waals forces via their aromatic cores, while their polar groups interact with the aqueous phase to create rigid interfacial films. These interfacial films inhibit droplet coalescence and stabilize emulsions. However, their hydrophobic nature (dominated by asphaltenes’ nonpolar frameworks) favors the stabilization of W/O emulsions. These factors collectively contribute to the viscosity elevation observed in the heavy crude oil emulsion system.

The relative concentration profiles of components in the DMS-containing system are displayed at 0 ps and 5000 ps, respectively (Figure 18a′,b′). Following a 5000 ps Number-Volume-Temperature Ensemble (NVT) ensemble simulation, DMS surfactant molecules spontaneously adsorb at the oil–water interface. The hydrophobic groups of DMS interact with asphaltenes and resins at the interface, while its sulfonic acid and carboxylic acid groups form hydrogen bonds with water molecules. The DMS molecules predominantly occupy the interfacial domain (Figure 18c′), displacing the original asphaltene-rich interfacial architecture (Figure 18c). The DMS-stabilized interfacial film (HLB = 11, indicative of pronounced hydrophilicity) preferentially stabilizes O/W emulsions, thereby inducing phase inversion in the heavy crude oil emulsion system and consequently reducing viscosity. Furthermore, the observation of smoother profiles in asphaltenes and resins in the relative concentration distribution curves suggests that the molecules are less tightly packed and more evenly dispersed. This finding indicates that the addition of DMS can effectively reduce the intermolecular interactions between asphaltenes and resins in the oil phase.

**Figure 19:**
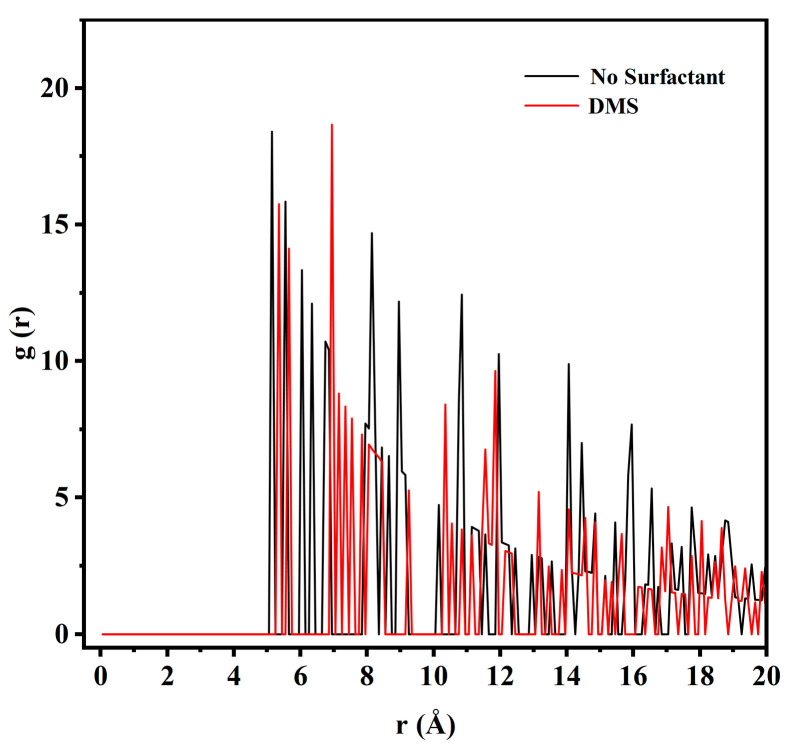
RDF curves of asphaltenes and resins at 5000 ps.

The validity of the proposed mechanism was further substantiated through radial distribution function (RDF) analysis. RDF curves provide an intuitive representation of the degree of particle aggregation and the likelihood of molecular interactions at specific distances. Figure 19 presents a comparison of the RDFs for asphaltenes and resins at 5000 ps in systems with and without surfactant, based on molecular dynamics simulations. The centroids of aromatic rings in asphaltenes and resins were selected as the calculation points. In the absence of surfactants, several consecutive peaks appeared around 5 Å–7 Å, indicating the presence of strong intermolecular interactions. These interactions most likely correspond to edge-to-face (T-shaped, π-σ) and offset π–π stacking (σ-σ) configurations [63]. Upon the introduction of DMS, both the number and intensity of peaks in this range decreased significantly. This reduction strongly suggests that DMS disrupts the π–π stacking and other non-covalent interactions among asphaltenes and resins, thereby inhibiting their aggregation. Consequently, the weakened molecular associations contribute to a reduction in the viscosity of the oil phase.

### 2.6. Emulsification Viscosity Reduction Mechanism of DMS Surfactant

**Figure 20:**
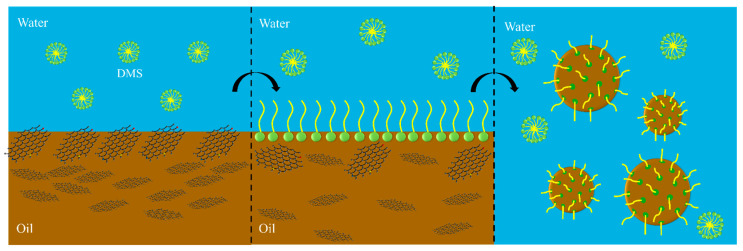
Mechanism of DMS viscosity reduction.

As demonstrated in Figure 20, the viscosity reduction mechanism of DMS in emulsions can be conceptualized. DMS, with an HLB value of 11, is a surfactant that favors the formation of O/W emulsions. Upon addition to the system, DMS rapidly adsorbs at the oil–water interface, displacing natural surfactants present in the heavy oil and forming a hydrophilic interfacial film. This process induces phase inversion from a W/O to an O/W emulsion. Concurrently, DMS enhances the absolute zeta potential of oil droplets, thereby effectively impeding droplet coalescence and stabilizing the dispersed system. In addition, the incorporation of DMS leads to a reduction in supramolecular interactions between asphaltenes and resins. This disruption of π–π stacking is believed to be a contributing factor to the observed decrease in oil phase viscosity [19,64]. The emulsifying properties of DMS are not confined to heavy oil emulsions; they also extend to the dispersion of resins and asphaltenes within the oil phase. The combined effect of these mechanisms is a synergy that leads to a significant reduction in emulsion viscosity.

## 3. Materials and Methods

### 3.1. Materials

Alkaline ligninAL (≥99.5 wt%) was supplied by Anhui Zesheng Technology Co. (Anqing, China) SDBS (≥95.0 wt%), and NaCl (≥99.5 wt%), CaCl_2_ (≥99.5 wt%), MgCl_2_ (≥99.5 wt%), NaHCO_3_ (≥99.5 wt%), and anhydrous ethanol (≥99.5 wt%) were purchased from Shanghai Maklin Biochemical Technology Co. (Shanghai, China) Hydrochloric acid (HCl, 36.0–38.0 wt%) was obtained from Sinopharm Chemical Reagent Co. (Shanghai, China) Potassium persulfate (K_2_S_2_O_8_, ≥99.5 wt%) and 3-((2-(methacryloxy)ethyl)dimethylammonium propane-1-sulfonate (≥98.0 wt%) were provided by Shanghai Titan Technology Co. (Shanghai, China) and Shanghai Bid Pharma Technology Co. (Shanghai, China), respectively. DMAPMA (≥98.0 wt%) was sourced from Shanghai Haohong Biomedical Technology Co. (Shanghai, China). Ultrapure water was obtained from an ECUST laboratory purification system. The Xinjiang heavy oil sample showed a dynamic viscosity of 1850 mPa·s at 50 °C and a density of 0.9601 g/cm^3^.

### 3.2. Synthesis of DMS Surfactant

**Scheme 1:**
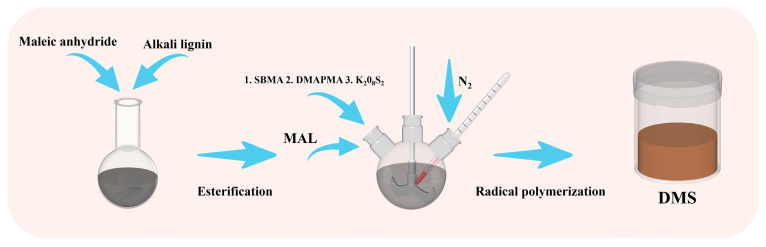
Synthesis of DMS surfactant.

The DMS surfactant was synthesized via a two-step procedure involving esterification, followed by free radical-mediated graft copolymerization (Scheme 1). For a comprehensive description of the synthesis method, please refer to the Supplementary Materials (Figure S3).

### 3.3. Characterization

The elemental composition (C, H, N, S) of DMS was quantified using a UNICUBE elemental analyzer (Elementar, Langenselbold, Germany), with oxygen content specifically determined via a dedicated detection mode. Molecular weight distribution was analyzed by gel permeation chromatography (GPC, Waters 1515, Milford, MA, USA) using ultrapure water as the mobile phase (flow rate: 1.00 mL/min; injection volume: 100.0 μL). FT-IR spectra were recorded on a Nicolet iS20 spectrometer (Thermo Fisher Scientific, Waltham, MA, USA) via a KBr pellet method across 400–4000 cm^−1^. ^1^H NMR analysis was performed using a Bruker Ascend 600 MHz spectrometer (Billerica, MA, USA) with D_2_O solvent. Surface chemical states were examined by X-ray photoelectron spectroscopy (XPS, K-Alpha, Thermo Scientific, Waltham, MA, USA) with adventitious carbon C1s calibration at 284.8 eV.

### 3.4. Surfactant Performance Evaluation Methods

#### 3.4.1. Hydrophilic–Lipophilic Balance Measurements

The HLB value of DMS surfactant was evaluated via an emulsion stability assay [40]. Turpentine (HLB 16) and cottonseed oil (HLB 6) were blended at varying ratios to generate standard oil mixtures covering HLB values from 7 to 16. Equal masses (20 g) of each oil sample and 0.1 wt% DMS solution were combined in centrifuge tubes, followed by manual agitation for 200 reciprocating cycles to achieve uniform emulsification. The mixtures were then left undisturbed to allow for complete phase separation between aqueous and oil layers. The time required for total water–oil separation was recorded, with the surfactant’s HLB value assigned to the oil sample demonstrating the longest stability period.

#### 3.4.2. Surface Tension Measurement

Surfactant solutions were prepared through serial dilution with ultrapure water to establish concentration gradients. Surface tension was measured at 25.0 °C using a JK99B tensiometer (Shanghai Zhongchen Co., Shanghai, China) employing the Du Noüy ring method. Prior to testing, all solutions were equilibrated at 25.0 °C for 30 min to ensure air–liquid interfacial adsorption equilibrium. For comparative purposes, SDBS, an anionic surfactant widely used in petroleum applications, was selected as a reference in evaluating the interfacial behavior of DMS.

#### 3.4.3. Salt Tolerance Measurements

To investigate the salt tolerance of the surfactant, its solubility in salt solutions containing varying concentrations of sodium (Na^+^) or calcium (Ca^2^^+^) ions was evaluated. The detailed experimental procedure is as follows: First, a stock solution of the surfactant was prepared using deionized water. Then, a series of NaCl and CaCl_2_ solutions with different concentrations were prepared to cover the full range of salt concentrations where precipitation might occur. All salt solutions were freshly prepared using deionized water to ensure accuracy and consistency. Subsequently, the surfactant stock solution was added to each salt solution to obtain a final surfactant concentration of 1 g/L. The mixtures were then subjected to ultrasonic treatment at 25.0 °C for 1 h to enhance the dispersion and solubility of the surfactant in the saline environment. After ultrasonication, each sample was visually inspected for signs of turbidity, precipitation, or phase separation. The appearance of visible precipitates or noticeable turbidity was taken as an indication that the surfactant had begun to precipitate, signifying that the salt concentration had reached or exceeded the surfactant’s salt tolerance limit. The lowest salt concentration at which precipitation or turbidity was first observed was defined as the salt tolerance threshold of the surfactant under the corresponding salt ion (Na^+^ or Ca^2^^+^) [40].

### 3.5. Emulsification and Viscosity Reduction Evaluation of Heavy Oil

#### 3.5.1. Preparation of Heavy Oil Emulsions

**Figure 21:**
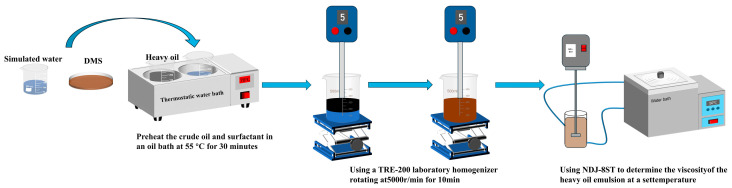
Schematic of the experimental setup.

The Xinjiang heavy oil and surfactant solution were preheated at 55 °C for 30 min. DMS surfactant solutions were prepared using synthetic formation water, as detailed in Table 4. Following preheating, the heavy oil and surfactant solution were mixed at an oil-to-water ratio of 6:4. Emulsification was then carried out using a TRE-200 homogenizer (Tianrui Experimental Equipment Co., Ruzhou, China) operating at 5000 rpm for 10 min, resulting in the formation of stable O/W emulsions [17]. A schematic of the experimental setup is provided in Figure 21 to visually represent the procedure.

#### 3.5.2. Determination of Viscosity-Reducing Effects of Emulsion

Emulsion viscosity was measured at 50 °C using an NDJ-8ST rotational viscometer (Shanghai Nirun Intelligent Technology Co., Shanghai, China). The viscosity reduction rate (f) was calculated as follows:

(1) $$f=\frac{\mu_{0}-\mu }{\mu_{0}}\times 100\%$$

where *μ*_0_ and *μ* represent the viscosities of neat heavy oil (mPa·s) and emulsion (mPa·s), respectively.

#### 3.5.3. Determination of Emulsion Stability

The configured oil–water emulsion was poured into a glass tube marked with a scale at 50 °C and was allowed to stand at a constant temperature. The amount of dewatering was recorded at regular intervals. The water separation rate was used to express stability, and the formula was calculated as shown in Equation (2).

(2) $$\varphi =\frac{V_{t}}{V_{0}}\times 100\%$$

where *φ* was the dewatering rate (%), *v_t_* was the volume of the separated water (mL), and *v*_0_ was the volume of the water in the emulsion (mL).

### 3.6. Emulsification and Viscosity Reduction Mechanism of DMS Surfactant

#### 3.6.1. Particle Size Distribution of Emulsions

The emulsion microstructure was characterized by using a Nikon E200 optical microscope (Tokyo, Japan) under bright-field illumination. Droplet size distribution analysis was performed using ImageJ 1.8.0 software. To ensure the attainment of representative results, statistical analysis was conducted at a minimum of 450 droplets per sample.

#### 3.6.2. Determination of Interfacial Tension (IFT)

The oil–water interfacial tension was measured using a TX500C rotating drop interfacial tension meter (USA KINO Scientific Instrument Inc., Boston, MA, USA). During the entire experiment test, the temperature was set to 50 °C. The test was completed when the oil–water interfacial tension data are stable.

#### 3.6.3. Determination of Zeta Potential (ξ)

The Zeta potential on the surface of oil droplets was measured using a JS94H micro—electrophoresis instrument (Shanghai Zhongchen Digital Technology Equipment Co., Ltd., Shanghai, China) at a temperature of 25 °C.

#### 3.6.4. Heavy Oil Rheological Properties

Rheological measurements of the oil–water emulsions were conducted using a HAAKE MARS 3 rheometer (Thermo Fisher Scientific, Karlsruhe, Germany). The viscosity–shear rate relationship of the heavy oil emulsions was determined at 50 °C under a strain of 5% (γ) and an angular frequency of 10 rad/s, with the shear rate ranging from 0.1 to 1000 s^−1^. Additionally, the modulus–frequency relationship was measured at 50 °C under a constant strain of 5%, with the frequency varying from 0.1 to 180 rad/s.

### 3.7. Molecular Dynamics Simulations’ Details

All molecular dynamics simulations were conducted using Materials Studio 6.1 with the COMPASS III force field. The initial molecular structures were constructed in the Visualizer module and geometrically optimized via the Forcite module. Two cubic simulation boxes (6 nm × 6 nm × 6 nm) were constructed: one containing water molecules, DMS surfactant, asphaltenes, resins, alkanes, naphthenes, and aromatics, and a control system with identical composition excluding DMS surfactant. Both systems underwent energy minimization under NPT ensemble (325 K, 1.0 atm) to achieve equilibrium density, followed by 5 ns NVT equilibration to stabilize thermodynamic parameters and establish interfacial adsorption equilibrium. Molecular models of asphaltenes, resins, and DMS are detailed in the Supplementary Materials (Table S1). Temperature and pressure were maintained using a Nose thermostat and a Berendsen barostat, respectively, throughout the simulations.

## 4. Conclusions

A lignin-based zwitterionic surfactant (DMS) was synthesized through a combination of esterification and free-radical-initiated graft copolymerization. The experimental results showed that DMS achieved a 91.6% viscosity reduction in heavy oil emulsion. Remarkably, it retained 84.4% efficiency at extreme salinity (TDS = 200,460 mg/L), overcoming the limitations of chemical flooding in high-salinity reservoirs. Mechanistic investigations have revealed that DMS reduces emulsion viscosity through two synergistic pathways: (1) inducing phase inversion from W/O to O/W emulsions via hydrophilic-lipophilic interface modification, and (2) disrupting asphaltene–resin aggregation in the oil phase to lower cohesive forces. The resulting DMS-stabilized O/W emulsion exhibits enhanced colloidal stability, evidenced by a zeta potential of −30.89 mV and reduced dispersed-phase droplet size (from 16.98 μm to 9.75 μm). This study provides a pioneering strategy for designing eco-friendly, salt-tolerant viscosity reducers for heavy oil applications, with potential implications for sustainable resource exploitation in high-salinity reservoirs.

## Figures and Tables

**Table 1:** Advances in surfactant research.

Surfactant	Crude Oil	Viscosity Reduction Rate (%)	References
Polyether carboxylates (APAD)	North China heavy oil.	98.34%	[14]
Gemini (E4A15)	Bohai oilfield heavy oil.	98.0%	[15]
Isooctyl glucoside (APG08)	Karamay heavy oil.	88.82%	[16]
Boron-containing anionic-nonionic surfactants (SYW)	Xinjiang heavy oil.	97.3%	[17]
Chitosan-based cationic surfactants (CBCS)	Western India heavy oil.	82%	[18]
Terpolymer of Bisphenol (AFOP-n)	Bohai Sea heavy oil.	98.22%	[19]
polyether carboxylic acid–sulfonic acid polymeric (SAAP)	North China heavy oil.	95%	[20]
Switchable oligomeric surfactants (OA/cyclen)	Shengli heavy oil.	99%	[21]

**Table 2:** Elemental composition analysis of DMS surfactant.

Element	C	H	O	S	N
Content (wt%)	57.30	9.29	21.16	3.10	9.17

**Table 3:** Molecular weight distribution of maleated alkali lignin (MAL) and DMS.

Sample	Mn (g/mol)	Polydispersity Index (PDI)
MAL	1031	2.88
DMS	3643	1.24

**Table 4:** Ion compositions of simulated formation water.

Ion Types	Cl^−^	HCO_3_^−^	Na^+^	Ca^2+^	Mg^2+^	TDS
Concentration (mg/L)	5863	341	3510	220	89	10,023

## Data Availability

Data are contained within this article or the Supplementary Materials.

## Supplementary Materials

The following supporting information can be downloaded at: https://www.mdpi.com/article/10.3390/molecules30112419/s1, Figure S1. GPC of surfactant: (a) MAL; (b) DMS. Figure S2. XPS wide survey spectra of DMS surfactant. Figure S3. Reaction equation of surfactant. Table S1. Molecular composition in the simulation box.
