# Supplementary material for: A Lignin-Based Zwitterionic Surfactant Facilitates Heavy Oil Viscosity Reduction via Interfacial Modification and Molecular Aggregation Disruption in High-Salinity Reservoirs
Source: Molecules. 2025 May 31;30(11):2419. doi: 10.3390/molecules30112419 (PMC12155679; doi:10.3390/molecules30112419)
Supplement: Supplementary file 1 [file molecules-30-02419-s001.zip › molecules-3650931-supplementary.pdf]

Supporting information

A Lignin-Based Zwitterionic Surfactant Facilitates Heavy Oil Viscosity Reduction via Interfacial Modification and Molecular Aggregation Disruption in High-Salinity Reservoirs

Qiutao Wu [1], Tao Liu [2], Xinru Xu [1] and Jingyi Yang [1,*]

[1] International Joint Research Center of Green Energy Chemical Engineering, East China University of Science and Technology, Meilong Road 130, Shanghai 200237, China.

[2] Shanghai Key Laboratory of Multiphase Materials Chemical Engineering, School of Chemical Engineering, East China University of Science and Technology, Shanghai 200237, China

***** Correspondence: jyyang@ecust.edu.cn

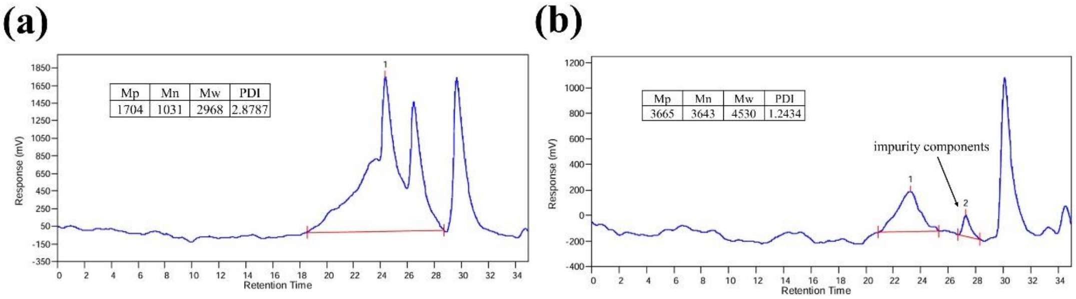

Figure S1. GPC of surfactant: (a)MAL; (b)DMS.

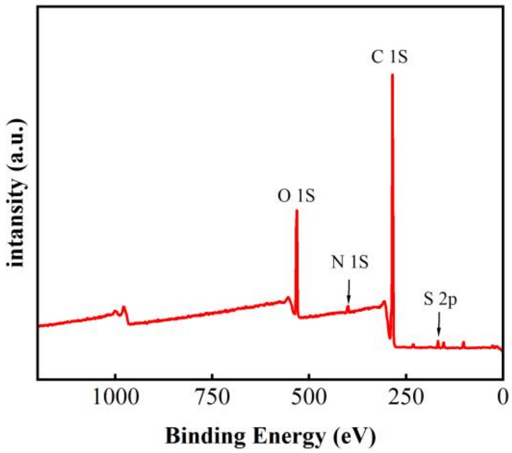

Figure S2. XPS wide survey spectra of DMS surfactant.

1.1 Purification of Lignin

The dispersion of alkali lignin (AL) in ultrapure water at a solid-to-liquid ratio of 1:10 (w/v) was conducted under magnetic stirring at 60°C for 2 h. Thereafter, the mixture was subjected to centrifugation at 2000 rpm for 60 min. The resultant pellet was collected, and the pH was adjusted to 2.0 using 1 M HCl. Following a period of 24 hours of static sedimentation, the precipitated lignin was separated by means of centrifugation. The precipitate was then subjected to repeated washing with ultrapure water until the filtrate reached a neutral pH, whereupon it was dried in a forced-air oven at 100°C for a period of 24 hours. The purified lignin was then ground and sieved through a 100-mesh sieve for subsequent use.

1.2 Synthesis of Maleated Lignin

In a suitable container, purified alkali lignin (10 g) was added to a molten maleic anhydride solution (50 g), which was maintained at 70 °C. The mixture was then stirred magnetically at 120 °C for 4 h. Following this, the mixture was allowed to cool to 60 °C, after which 200 mL of absolute ethanol was added with the intention of precipitating the product. The product was then washed thrice with an ethanol–water mixture (3:1, v/v) and dried under vacuum at 60℃ for 24 h to obtain maleated alkali lignin (MAL), which was subsequently stored in a desiccator. The synthesis route is shown in Scheme 1.

1.3 Synthesis of DMS Surfactant

MAL (10 g) was dispersed in 70 mL of ultrapure water, and the pH was adjusted to 9.0 using 1 M NaOH.A series of aqueous solutions were then prepared: solutions of SBMA (20 wt%) and DMAPMA (20 wt%), along with a 10 wt% potassium persulfate initiator solution. Under nitrogen protection, the MAL solution, monomer solutions (molar ratio MAL:SBMA:DMAPMA = 1: 3.9: 5.8), and $K_2S_2O_8$ (1.5 % of the total monomer mass) were sequentially added to a four-necked flask equipped with a stirrer and thermometer. The reaction was then carried out at 65℃ for 6 hours. The product was purified by dialysis (molecular weight cutoff: 1000 Da, N-Buliv) for 3 days, with water replaced every 6 hours. After drying, a brown solid was obtained. The synthesis route is shown in Scheme 2.

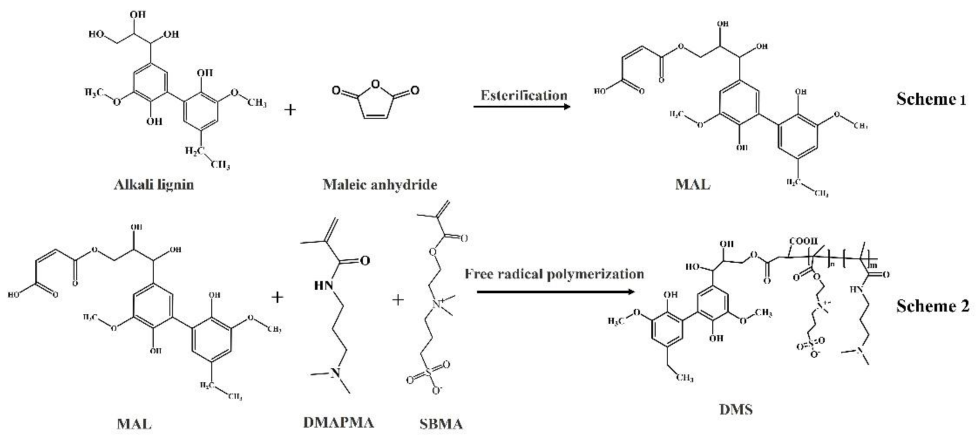

Figure S3. Reaction equation of surfactant.

Table S1. Molecular composition in the simulation box

Name	CAS Number	molecule	Number of molecules
Asphalt 1		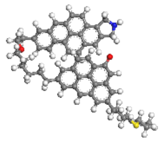	1
Asphalt 2		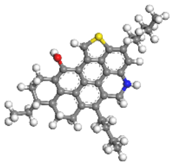	2
Resin 1		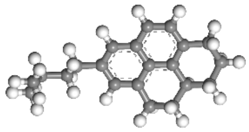	10
Resin 2		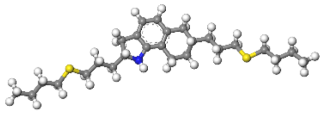	9
Resin 3		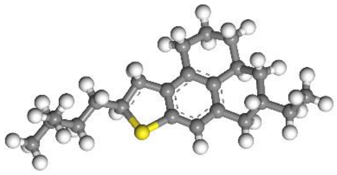	8
DMS		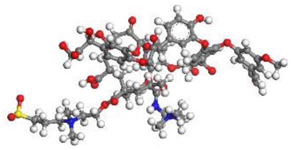	4
Water		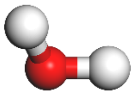	3716
1-ethyl-4-methylbenzene	622-96-8	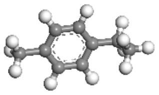	66
Methylnaphthalen	1321-94-4	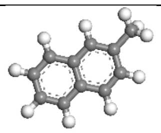	86
Cyclohexane	110-82-7	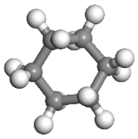	34

Hexyl hydride	110-54-3	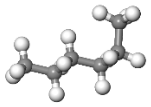	53
Methylcyclohexane	108-87-2	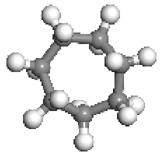	41
3-Ethylpentane	617-78-7	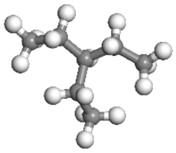	38
2,4-Dimethylhexane	589-43-5	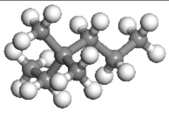	50
3,3-Dimethylheptane	4032-86-4	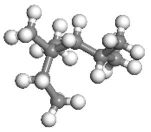	63
